# Molecular Detection and Characterization of *Cryptosporidium* species in Chickens From Commercial Farms and Live Bird Markets in Ibadan, Nigeria

**DOI:** 10.1155/vmi/3818821

**Published:** 2026-09-09

**Authors:** Martina Oluwatimileyin Oyedepo, Omolade A. Oladele, Adetolase A. Bakre, Olayinka Remilekun Anifowose, Foluke Adedayo Akande, Opeyemi K. Olofintuyi, Ridwan Olamilekan Adesola

**Affiliations:** ^1^ Department of Animal Health Technology, Federal College of Agriculture, Ibadan, Oyo, Nigeria; ^2^ Department of Veterinary Medicine, University of Ibadan, Ibadan, Oyo, Nigeria, ui.edu.ng; ^3^ Department of Veterinary Parasitology and Entomology, College of Veterinary Medicine, Federal University of Agriculture, Abeokuta, Nigeria, mouau.edu.ng

**Keywords:** *Cryptosporidium*, molecular detection, Nigeria, poultry, zoonosis

## Abstract

**Background:**

Cryptosporidiosis is an important intestinal disease of poultry with zoonotic implications. However, routine molecular surveillance remains limited in Nigeria’s commercial poultry sector. This study aimed to detect and characterize *Cryptosporidium species* in commercial chickens and live bird markets (LBMs) in Ibadan, Oyo State, using microscopy and molecular methods.

**Materials and Methods:**

A total of 179 pooled dropping samples were collected from commercial farms and LBMs across eight sampling locations in seven local government areas. Samples were examined using modified Ziehl–Neelsen staining and processed by nested PCR targeting the 18S rRNA gene.

**Results:**

Microscopy revealed an overall detection rate of 18.99% (34/179). Prevalence was higher in commercial farms (21.13%) than in LBMs (10.81%). Layers showed greater infection (23.17%) than broilers (15.46%), with birds aged 21–40 weeks most affected. Of the 34 microscopy‐positive samples, five (14.70%) were PCR‐confirmed: *Cryptosporidium baileyi* (4/34; 11.76%) and the zoonotic *C. meleagridis* (1/34; 2.94%).

**Conclusions:**

The detection of zoonotic *C. meleagridis* highlights a public health risk. These findings emphasize the need for enhanced biosecurity, targeted management practices, molecular surveillance, and farmer awareness to reduce cryptosporidiosis in poultry systems.

**Highlights**
• Cryptosporidium infection was detected in chickens from commercial farms and LBMs in Ibadan, Nigeria.
• PCR confirmed the presence of *Cryptosporidium baileyi* and the zoonotic *Cryptosporidium meleagridis.*
• Detection of zoonotic *C. meleagridis* indicates a potential public health risk associated with poultry systems.

## 1. Introduction

Poultry production plays an important role in the agricultural economy of Nigeria, contributing significantly to food security and income generation. It accounts for approximately 25% of locally produced meat [1] and serves as a major source of animal protein for the country’s growing population [2]. With the rapid expansion of commercial poultry systems, especially under intensive management, the risk of infectious diseases has escalated, posing a serious challenge to optimal productivity [3–7]. One such disease is cryptosporidiosis, a protozoan infection that often goes undiagnosed or underestimated yet can severely impair poultry performance and welfare [8].

*Cryptosporidium species* is an enteric parasite with a wide host range, infecting mammals, birds, reptiles, and fish [9]. In avian species, the parasite was first identified in the caeca of chickens [10] and has since been reported in over 30 bird species globally, including ducks, turkeys, geese, quails, and pheasants [11]. The genus belongs to the same taxonomic group as other coccidian protozoa such as *Toxoplasma*, *Eimeria*, and *Isospora* [12], but it is distinguished by its unique life cycle, marked by the shedding of double‐walled, sporulated oocysts that contain infective sporozoites [12]. Among the 31 known *Cryptosporidium* species, four are primarily associated with birds: *C. meleagridis*, *C. baileyi*, *C. galli*, and *C. avium* [13]. Of these, *C. baileyi* is most frequently detected in domestic poultry, causing respiratory and intestinal lesions, particularly in the bursa of Fabricius. *C. meleagridis*, while mainly intestinal, is of zoonotic concern, implicated in infections of immunocompromised individuals and young children [14]. Transmission occurs via ingestion or inhalation of sporulated oocysts found in contaminated feces, water, dust, and bedding [15]. Poor hygiene and sanitation exacerbate the spread, especially in overcrowded poultry environments [16]. The risk of zoonotic transmission is heightened by the presence of *C. meleagridis* in poultry species and the reported reverse zoonosis of *C. parvum*, a common species in humans and livestock, from humans to animals [17].

In Nigeria, *Cryptosporidium species* pose a growing threat to poultry farming, particularly in regions such as Ibadan [18], where intensive poultry operations and live bird markets (LBMs) are common. Factors such as age, land use, and seasonal variation influence infection rates [19], and the disease is likely underdiagnosed due to symptom overlap with other protozoan diseases such as coccidiosis [15]. Clinical signs in infected birds include diarrhea, weight loss, decreased feed intake, and impaired growth, all contributing to economic losses through reduced egg and/or meat yield and increased management costs [10, 20]. Also, the public health significance of *Cryptosporidium species* has come under scrutiny due to its zoonotic potential. The detection of *C. parvum* Subtype IIc in human cases in Nigeria highlights the role of anthroponotic transmission [18]. Nonetheless, studies exploring the molecular epidemiology of *Cryptosporidium* species in Nigerian poultry are sparse. Enhanced diagnostic tools such as polymerase chain reaction (PCR) can improve sensitivity and specificity, enabling early and accurate detection, especially in asymptomatic or immunocompromised birds [21]. Given these challenges, this study aimed to determine the prevalence, circulating species, and genetic detection of *cryptosporidium species* in chickens from commercial farms and LBMs in the Ibadan metropolis of Oyo State.

## 2. Materials and Methods

### 2.1. Study Location

The study was carried out in Ibadan, the capital of Oyo State, Nigeria. Ibadan is located between latitudes 7°20′N and 7°40′N and longitudes 3°30′E and 4°10′E, covering an area of approximately 129.65 km^2^. With a population exceeding 3.5 million, it is Nigeria’s third most populous city and the largest by land area [22]. Economically, the city is a key agricultural and commercial hub, known for the trade of cassava, cocoa, palm oil, and timber. It also hosts numerous cattle ranches, abattoirs, and commercial poultry farms, making it suitable for studying poultry‐related infections.

### 2.2. Study Design

Using a multistage sampling method along with a cross‐sectional design, *Cryptosporidium species* were detected in chickens from commercial poultry farms and LBMs across eight sampling locations in seven local government areas (LGAs) in the Ibadan metropolis. Farms were selected from Akinyele, Egbeda, Ido, Lagelu, and Oluyole LGAs, while markets were sampled from Ido, Ibadan South‐East, and Ibadan North LGAs. Fecal samples were collected by pooling freshly voided droppings from three birds into a single composite sample. Pooling was employed to increase the likelihood of detecting low‐level infections within a pen while remaining cost‐effective; prevalence estimates reported in this study therefore reflect the proportion of pooled samples testing positive rather than individual bird‐level prevalence. Chickens were categorized into four age groups for analytical purposes: 1–20 weeks, 21–40 weeks, 41–60 weeks, and above 60 weeks. Pooled dropping samples were individually identified and assigned codes using the location and date of collection before they were transported in an icebox to the laboratory for further analysis.

### 2.3. Sample Size Determination

The sample size was calculated using the formula outlined by Thrusfield [23]. Given the absence of prior molecular studies on *Cryptosporidium* in chickens in the Ibadan metropolis, the nearest available prevalence estimate of 20.6% was reported by El‐Bakri et al. [21], while the confidence interval was set at 95% and the precision at 5%. An estimated 179 samples were collected.

### 2.4. Collection of Dropping Samples

Standard physical restraint was ensured to prevent injury and reduce stress and pain elicitation during sampling. Using sterile gloves, an ample amount of droppings was collected from the cloaca of the birds, put into sterile plain bottles, and transported in cool boxes to the laboratory. Prior to sampling, flock health status was assessed through owner interviews and direct observation. Chickens on commercial farms were apparently healthy, with laying flocks maintaining normal egg production and no reported history of vaccination failure. All flocks had updated vaccination records, mortality rates were minimal, and postmortem examination of dead birds revealed no pathognomonic lesions consistent with cryptosporidiosis. Birds presented at LBMs were also visually healthy and fit for sale.

### 2.5. Detection of *Cryptosporidium* Oocysts by Microscopy

Each pooled fecal sample (∼ 1 g) was homogenized in distilled water using a mortar and pestle, sieved, and centrifuged at 2500 g for 10 min. The sediments were used to prepare thin smears on clean microscope slides, which were then air‐dried, fixed in absolute methanol for 5 min, and stained with carbol fuchsin for another 5 min. Slides were rinsed and then decolorized with 3% acid alcohol for 15 min (3% HCl in ethanol), counterstained with 0.5% malachite green for 1 min, and rinsed out before air‐drying. Microscopic examination for *Cryptosporidium oocysts* was performed using oil immersion at × 100 magnification [20]. Remaining samples from the sediment were stored at −4°C for molecular analysis.

### 2.6. DNA Extraction and PCR Amplification

Genomic DNA was extracted from the sediment of positive pooled dropping samples using a modified phenol–chloroform protocol [24, 25]. Species‐specific primers targeting 18 rRNA were used to detect *C. meleagridis* and *C. baileyi* at expected band sizes of 792 and 882 bp, respectively. PCR reactions were prepared in a 50 µl mixture containing 21.6 µl nuclease‐free water, 25 µl of master mix (Roche, USA), 1.2 µl each of forward and reverse primers, and 1 µl of DNA template (Table 1). *Cryptosporidium hominis* and nuclease‐free water were used as positive and negative controls, respectively. Fully Nested PCR reactions were set up in a 20 µl volume using Maxima Hot Start PCR Master Mix (Thermo Scientific), with amplification carried out as follows: initial denaturation at 95°C for 4 min, 35 cycles of denaturation at 95°C for 30 s, annealing at 60°C (primary PCR) or 58°C (nested PCR) for 30 s, extension at 72°C for 1 min, and a final extension at 72°C for 7 min. PCR products were visualized on 1.5% agarose gels stained with ethidium bromide.

**TABLE 1:** Primers used for the amplification of *Cryptosporidium* species.

Species	Reaction	Primer	Reference	Expected band size
*C. meleagridis*	PCR	F: GATGAGATTGTCGCTCGTTATC	[26]	792 bp
		R: AACCTGCGGAACCTGTG		
	Nested	F: GAGATTGTCGCTCGTTATCG		
	PCR	R: GATTCAAAAACGGAAGG		

C. *baileyi*	PCR	F: GACATATCATTCAAGTTTCTGACC	[27]	882 bp
		R: CTGAAGGAGTAAGGAACAACC		
	Nested	F: CCTATCAGCTTTAGACGGTAGG		
	PCR	TCTAAGAATTTCACCTCTGACTG		

### 2.7. Statistical Analysis

Descriptive statistics and chi‐square tests were used to analyze the prevalence of *Cryptosporidium* species across different sample sources and bird types using SPSS Version 23. A *p* value of < 0.05 was considered statistically significant [28].

## 3. Results

An overall detection rate of 18.99% was revealed. Commercial farms had a higher detection rate (21.13%, 30/142) than LBMs (10.81%, 4/37) (Table 2). Ido LGA had the highest detection rate among the commercial farms examined across five LGAs (27.27%, 9/33), followed by Akinyele (23.53%, 8/34), Egbeda and Lagelu (20.00% each, 3/15 and 6/30, respectively), and Oluyole (13.33%, 4/30) (Table 2). The detection rates from the LBMs were 0% (0/5) in Apata (Ido LGA), 22.22% (2/9) at Gate (Ibadan North), and 8.70% (2/23) at Molete (Ibadan South‐East) (Table 2).

**TABLE 2:** Detection of *Cryptosporidium* species from farms and live bird markets in Ibadan.

Features	Samples	Positive (%)	OR (95% CI)	*p* value
Location (regions)				
Commercial farms	142	30 (21.1)	1	
Live bird markets	37	4 (10.8)	0.5 (0.2–1.4)	0.24
Location (local government)				
Ido	33	9 (27.3)	1	
Akinyele	34	8 (23.5)	0.8 (0.3–2.5)	0.78
Egbeda	15	3 (20)	0.7 (0.2–2.9)	0.73
Lagelu	30	6 (20)	0.7 (0.2–2.2)	0.56
Oluyole	30	4 (13.3)	0.4 (0.1–1.5)	0.22
Molete	23	2 (8.7)	0.3 (0.1–1.3)	0.1
Gate	9	2 (22.2)	0.8 (0.1–4.4)	1
Apata	5	0 (0)	0.2 (0.0–4.7)	0.3
Chicken types				
Broiler	97	15 (15.5)	0.6 (0.3–1.3)	0.25
Layer	82	19 (23.2)		
Age groups				
1–20	88	8 (9.1)	0.8 (0.2–3.5)	0.73
21–40	12	3 (25)	1	
41–60	42	9 (21.4)	0.8 (0.2–3.7)	1
> 60	37	4 (10.8)	0.4 (0.1–1.9)	0.34
Total	179	34 (18.9)		

### 3.1. Detection of *Cryptosporidium* in Different Chicken Types and Age Groups

A total of 97 broilers and 82 layers were examined. Of these, 15 broilers (15.46%) and 19 layers (23.17%) tested positive for *Cryptosporidium* under microscopy (Table 2). Based on the age categorization described in the methodology, 88 chickens between 1 and 20 weeks were examined, of which 18 (20.45%) were positive. Among 12 chickens aged 21–40 weeks, 3 (25.00%) were positive; among 42 chickens aged 41–60 weeks, 9 (21.43%) were positive; and among 37 chickens older than 60 weeks, 4 (10.81%) were positive (Table 2).

### 3.2. Microscopy, DNA Extraction, and PCR Analysis

**FIGURE 1:**
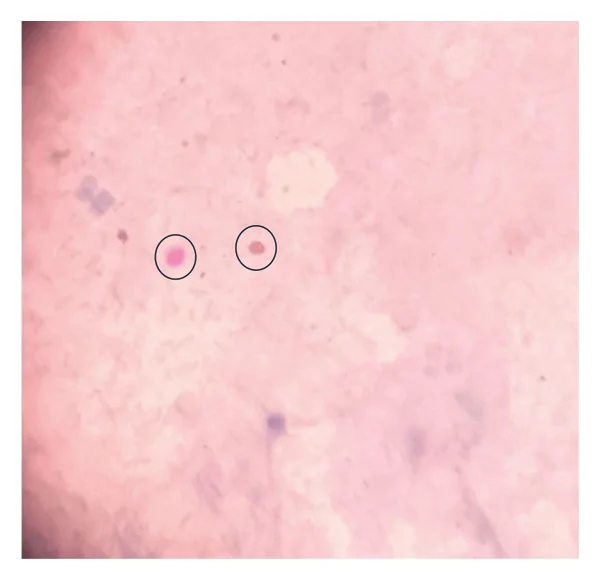
*Cryptosporidium oocysts* (indicated in circle) in dropping smears stained with Ziehl–Neelsen stain at × 100 magnification.

**FIGURE 2:**
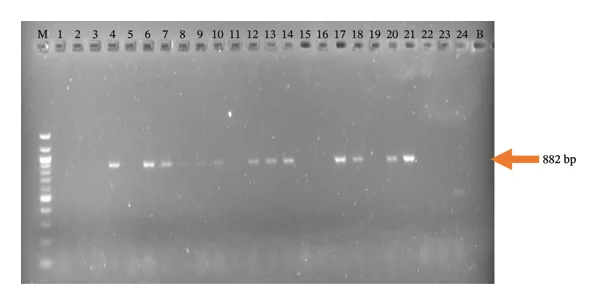
Agarose gel electrophoresis showing the positive amplification of the PCR products of the *Cryptosporidium baileyi* gene from chicken droppings in Ibadan. Lane M‐DNA ladder; lane 1‐24‐18S rRNA gene; ladder size: 1 kbp DNA fragment; expected band size approximately 882 bp.

**FIGURE 3:**
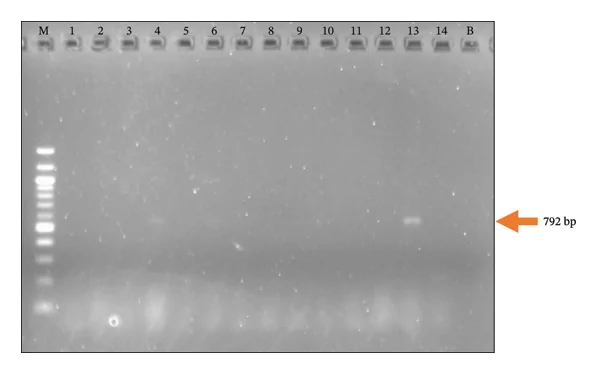
Agarose gel electrophoresis showing the positive amplification of the PCR products of the *Cryptosporidium meleagridis* gene from chicken droppings in Ibadan. Lane M‐DNA ladder; lane 1‐14‐18S rRNA gene; ladder size: 1 kbp DNA fragment; expected band size approximately 792 bp.

Out of the 34 microscopy‐positive samples, only 5 were confirmed by nested PCR targeting the 18S rRNA gene. Four of these samples (11.76%) and one (2.94%) were identified as *C. meleagridis* and *C. baileyi*, respectively. On Ziehl–Neelsen‐stained smears submerged in oil, *Cryptosporidium* oocysts showed up as brilliant red, oval‐shaped, acid‐fast organisms (Figure 1). Using agarose gel electrophoresis, positive amplification for *C. meleagridis* (792 bp) and *C. baileyi* (882 bp) was detected via agarose gel electrophoresis (Figures 2 and 3).

### 3.3. Discussion

This study investigated the prevalence and molecular characterization of *Cryptosporidium* species in chickens from commercial farms and LBMs in Ibadan Metropolis, Oyo State, Nigeria. An overall detection rate of 18.99% was observed, indicating that nearly one in five pooled dropping samples harbored *Cryptosporidium* oocysts. This prevalence is lower than the 28.7% reported by NO et al. [29] in Enugu State, but higher than 11.9% [1] in Akure and 4.85% [30] in Kano. Differences in detection rates across studies may be attributed to geographic variation, climatic conditions, sampling design, and diagnostic methods [18, 31]. Higher humidity, for instance, may promote oocyst survival, as likely in Enugu, while arid conditions such as those in Kano may limit transmission [32]. Other factors, including seasonality, host age, management practices, and biosecurity levels, further influence detection rates [33].

The difference in detection between commercial farms (21.13%) and LBMs (10.81%) highlights the role of poultry management systems. While farms promote intensive production, they may also inadvertently support pathogen spread through shared litter, water, equipment, and inadequate waste disposal. In contrast, the transient nature of bird housing in LBMs, where turnover is rapid, may reduce exposure time and pathogen accumulation [34]. Nonetheless, some LBMs such as Gate (Ibadan North) exhibited higher detection (22.22%), likely due to poorer hygiene, higher‐risk sourcing, lack of standardized sanitation protocols, and improper waste management, while others, such as Apata (Ido LGA), showed no detection, possibly due to cleaner conditions or small sample size.

Within commercial farms, detection rates varied across LGAs, with Ido (27.27%) and Akinyele (23.53%) recording the highest values. These locations may represent higher‐risk zones due to dense poultry farming clusters, poor sanitation, and proximity to mixed livestock systems that serve as reservoirs for *Cryptosporidium* [35]. In contrast, Oluyole LGA, which recorded the lowest rate (13.33%), may benefit from better infrastructure, veterinary oversight, and biosecurity due to its more urbanized status. Improved awareness and access to veterinary services may contribute to reduced infection rates [8].

Higher detection rate in layers (23.17%) compared to broilers (15.46%) corroborates earlier findings by Laatamna et al. [33] and Helmy et al. [9], who reported increased vulnerability in layers. Layers have a longer production cycle, increasing their cumulative exposure to oocyst‐contaminated feed, water, and equipment [12]. In addition, physiological stress during egg production and housing transitions can impair immune responses [36]. Broilers, in contrast, have shorter lifespans, often limiting detectable infection before slaughter [15]. Importantly, the higher detection observed during peak production is of great concern, as increased oocyst shedding at this stage may contribute to environmental contamination with significant public health implications, including washing into water bodies, use of poultry waste in fish farming, or application as organic fertilizer.

Age‐specific detection patterns showed the highest rate in birds aged 21–40 weeks (25.00%), a critical period coinciding with early egg‐laying. This phase is marked by stress and environmental changes, which may increase susceptibility to infection [37, 38]. Detection decreased in birds over 60 weeks (10.81%), likely due to acquired immunity or selective culling of less resilient individuals [39]. These findings underscore the chronic and recurrent nature of *Cryptosporidium* infections in poorly managed systems.

Only five out of the 34 microscopy‐positive samples were confirmed positive by PCR, illustrating the limitations of microscopy in species identification and sensitivity [40]. Molecular tools are thus essential for accurate surveillance. Of the PCR‐positive samples, four were identified as *C. baileyi* and one as *C. meleagridis*. *C. baileyi*, a common avian pathogen, is known to infect both respiratory and gastrointestinal tracts [9, 41]. Its presence across age groups and farm locations indicates widespread endemicity and environmental contamination.

The detection of *C. meleagridis*, although less frequent, raises significant public health concerns. As one of the few avian *Cryptosporidium* species known to infect humans, it poses zoonotic risks, especially in farm settings with poor hygiene [42, 43]. Its occurrence in this study supports previous reports of zoonotic transmission potential in avian hosts [8, 18].

## 4. Conclusion and Future Directions

This study documents the occurrence and molecular identity of *Cryptosporidium* species in poultry in Ibadan, Nigeria, providing the first evidence of *C. baileyi* and the zoonotic *C. meleagridis* in this setting. The findings establish poultry as a potential reservoir of cryptosporidiosis with relevance to both animal production and public health. It also emphasizes the need for integrated surveillance systems and improved farm hygiene, and they establish a foundation for future epidemiological and molecular studies to guide control strategies in Nigeria and beyond. Improving the control of *Cryptosporidium* infections in poultry requires a multifaceted approach that addresses both animal and public health. Ensuring that farm workers and bird handlers consistently use protective equipment such as gloves and face masks, alongside the promotion of good hygiene and proper waste disposal, can significantly reduce the risk of zoonotic transmission, particularly in settings with close human–poultry contact. Management practices should also be tailored to the specific needs of poultry types and age groups, with greater attention paid to birds in the early laying phase. Biosecurity measures in LBMs need to be strengthened through better sanitation, regulated bird sourcing, reduced bird holding times, and consistent health monitoring. In addition, raising public awareness and improving farmer education through community outreach, training programs, and locally relevant informational campaigns can empower poultry stakeholders to implement and sustain preventive measures. Expanding molecular and phylogenetic surveillance is equally important, as it will aid in tracking circulating *Cryptosporidium* strains, uncover transmission pathways, and support the development of targeted interventions, including potential vaccines and therapeutics suitable for the local poultry industry.

**Nomenclature**
LGA: Local government area
PCR: Polymerase chain reaction
RNA: Ribonucleic acid
DNA: Deoxyribonucleic acid

## Author Contributions

Omolade A. Oladele: writing–review and editing of the original draft. Martina Oluwatimileyin Oyedepo and Opeyemi K. Olofintuyi: software, methodology, investigation, formal analysis, and conceptualization. Adetolase A. Bakre: writing–review and editing the original draft, methodology, validation, conceptualization, formal analysis, and writing–review and editing the manuscript. Foluke Adedayo Akande and Olayinka Remilekun Anifowose: methodology, visualization, validation, and writing–review and editing of the manuscript. Ridwan Olamilekan Adesola: writing–review and editing the manuscript.

## Funding

The authors received no financial support for the research, authorship, and/or publication of this article.

## Disclosure

All authors reviewed the manuscript before final submission.

## Ethics Statement

This study was conducted following approval from the Animal Care and Use Research Ethics Committee (ACUREC) of the University of Ibadan, Nigeria, with approval number: NHREC/UIACUREC/05/12/2022A. Verbal informed consent was obtained from all poultry farm owners and live bird market vendors prior to sample collection.

## Conflicts of Interest

The authors declare no conflicts of interest.

## Data Availability

The data that support the findings of this study are available on request from the corresponding authors. The data are not publicly available due to ethical restrictions.
